# Prediction of aspiration in dysphagia using logistic regression: oral intake and self-evaluation

**DOI:** 10.1007/s00405-019-05687-z

**Published:** 2019-10-19

**Authors:** Bas J. Heijnen, Stefan Böhringer, Renée Speyer

**Affiliations:** 1 Department of Otorhinolaryngology and Head and Neck Surgery, Leiden University Medical Centre, Zone H2-Q, PO Box 9600, 2300 Leiden, RC The Netherlands; 2 Department of Medical Statistics, Leiden University Medical Centre, Leiden, The Netherlands; 3 Department of Special Needs Education, University of Oslo, Oslo, Norway; 4 School of Occupational Therapy and Social Work, Faculty of Health Sciences, Curtin University, Perth, Australia

**Keywords:** Swallowing disorders, Aspiration, Deglutition, Assessment, Predictive model, Logistic regression

## Abstract

**Objectives:**

Oropharyngeal dysphagia (OD) has a major influence on health in general and health-related quality of life (HR-QoL) in particular. The gold standard assessments for OD, especially for aspiration in OD, are fiberoptic endoscopic evaluation of swallowing (FEES) and videofluoroscopy (VFSS), but not all patients have access to such procedures. Therefore, the current study built a prediction model to forecast aspiration in patients with OD on the basis of common self-evaluation questionnaires and oral intake status.

**Methods:**

A consecutive series of 111 patients with confirmed diagnosis of OD was measured according to a standardised protocol using the following tools: the Swallowing Quality of Life Questionnaire (SWAL-QOL), the Dysphagia Handicap Index (DHI), two self-report visual analogue scales which measure the Severity and the Impairment of the swallowing problem on everyday social life as experienced by the patient, the Eating Assessment Tool 10 (EAT-10), the Functional Oral Intake Scale (FOIS) and subsequently FEES (the gold standard). Penalised logistic regression was carried out to predict aspiration. The performance of the resulting models was evaluated by constructing receiver operating characteristics (ROC) curves and computing areas under the curve (AUC).

**Results:**

The final model showed an AUC of 0.92, indicating excellent performance.

**Conclusion:**

This study shows that it may be possible to accurately predict aspiration in oropharyngeal dysphagia by a non-invasive and non-instrumental assessment protocol including oral intake status and self-report questionnaires on functional health status and HR-QoL.

## Introduction

Oropharyngeal dysphagia (OD) has a major influence on health in general and notably on health-related quality of life (HR-QoL) [1–3].

Aspiration or silent aspiration in severe OD can cause aspiration pneumonia and, when accompanied by malnutrition and dehydration, can lead to hospitalization, intensive care admission or even death [2, 4]. The gold standards for detecting aspiration and silent aspiration are videofluoroscopic (VFSS) and fiberoptic endoscopic evaluation of swallowing (FEES). While both have a high sensitivity and specificity [5], they are invasive, may be burdensome for the patient and are expensive. Moreover, these gold standards are not generally available in clinical settings such as a nursing home or general practice.

Screening for OD can be performed in various ways such as by trial swallows using water or substances with different viscosities, by oxygen desaturation or by cough elicitation [6, 7]. Screening should be sufficiently sensitive and specific but also easy to administer without extensive training [6]. When patients fail the screening, further assessment of OD is recommended. Numerous assessments are available to evaluate OD in further detail. Each one is focussed on certain domains such as functional health status (FHS), health-related quality of life (HR-QoL) or oral intake.

The presence of aspiration is the most critical clinical sign in patients with OD. However, the literature [8, 9] reveals a moderate to low correlation between self-evaluation questionnaires and aspiration as determined by FEES or VFSS. Also, oral intake as assessed by the FOIS shows weak correlations with aspiration [10]. One study [11] determined the accuracy of the EAT-10 using VFSS as reference test, to detect aspiration in patients at risk of OD; although sensitivity was sufficient, low specificity was found (respectively, 83% and 25%).

Predictive modelling entails developing a mathematical tool that generates an accurate prediction [12]. Several studies on dysphagia have used predictive modelling to forecast swallowing problems based on various criteria: for example, dosimetric parameters in radiotherapy [13, 14], tumour size and location [14], VFSS parameters [15] or cervical auscultation [16]. The predicted outcome of the models ranged from radiotherapy-induced dysphagia [13, 14] to persistent dysphagia after stroke [15] and presence of aspiration [16]. The predicted outcome of most studies was dysphagia, though not differentiating between dysphagia with or without aspiration. To the best of our knowledge, no models thus far have used individual or combined self-evaluation questionnaires on FHS and/or HR-QoL in OD for predicting aspiration.

The purpose of this study was to build a predictive model that could forecast aspiration in patients with OD using oral intake status and commonly used self-evaluation questionnaires on FHS and HR-QoL.

## Methods

All procedures performed in studies involving human participants were in accordance with the ethical standards of the institutional and/or national research committee and with the 1964 Helsinki declaration and its later amendments or comparable ethical standards.

### Patients

This study included a consecutive series of patients with OD during their first visit to the outpatient clinic of the department of Otorhinolaryngology and Head and Neck Surgery of the Leiden University Medical Centre. Patients were included if they (1) were at least 18 years old and (2) were not suffering from severe cognitive problems. Patients had sufficient communication skills for participating in daily conversation and completion of questionnaires (basic, comprehensive reading and writing skills). All had a confirmed diagnosis of OD based on FEES examination by an experienced ENT specialist or speech–language pathologist (SLP).

### Measures

FEES was performed in all patients as part of standard care at the outpatient clinic of the department of Otorhinolaryngology and Head and Neck Surgery. All patients completed several self-evaluation questionnaires during the week prior to their FEES exam. The following standardised protocols were used (listed in order of administration).

1. The SWAL-QOL [17–19] is a 44-item questionnaire on HR-QoL. It is considered to be the gold standard for measuring HR-QoL in OD [20]. The SWAL-QOL consists of ten subscales (Burden, Eating duration, Eating desire, Food selection, Communication, Fear, Mental health, Social functioning, Fatigue and Sleep) and one symptom scale (14 items, among which coughing, choking, gagging and drooling) [21]. The minimum and maximum scores on each subscale range from 0 to 100: not impaired to extremely impaired HR-QoL, respectively.
2. The DHI [22] measures FHS as well as HR-QoL. This 25-item questionnaire concerns the effect of OD on Physical (9 items), Functional (9 items) and Emotional (7 items) aspects of patients’ lives. Each item is scored as 0, 2 or 4, with higher scores meaning more severe disability. The total score ranges from 0 to 100. The DHI has one additional question about the severity of a patient’s swallowing problem ranging from 1–7 (Severity question: 1 normal, 7 severe problem).
3. Each of two concise self-report 100-mm Visual Analogue Scales (VAS) [23] measures a certain aspect of swallowing. One concerns the severity of the swallowing problem as experienced by the patient (Severity: FHS), whereas the other measures the perceived impact of the swallowing problem on everyday social life (Impairment: HR-QoL). Higher scores indicate greater impairment (range 0–100).
4. The EAT-10 [24] is a short 10-item self-administered questionnaire [7]. Although predominantly regarding FHS, it also includes some HR-QoL items [25]. Each one is rated on a five-point scale (0–4); the summed score ranges from 0 to 40 (higher scores are more abnormal). A sum of ≥ 2 [11] or ≥ 3 [24] is considered abnormal.
5. The Functional Oral Intake Scale (FOIS) [10] registers actual oral intake. The scores range from 1 (nothing by mouth) to 7 (total oral diet with no restrictions). During a patient’s first visit to the outpatient clinic (prior to expert advice on oral intake), the FOIS was completed by the clinician.

Subsequently, FEES was performed according to a standardised protocol [26]. Patients were offered three swallow trials of three different consistencies (nine trials maximum): methylene blue-dyed water (thin) or applesauce (thick) in portions of 10 mL and three bite-sized crackers with a fixed weight of 3.3 g (solid). In the event of aspiration, the trial of that particular consistency was stopped. FEES examinations were performed with a XION chip-on-the-tip flexible nasendoscope (Berlin, Germany). Results were recorded with RVC Clinical Assistant (Baarn, the Netherlands), a medical archive and image viewer. Recordings were rated by consensus among two SLPs using the Penetration Aspiration Scale (PAS) [27]. FEES results were dichotomised into no aspiration (PAS score 1–5) or aspiration (PAS scores 6–8) [28]. If the patient aspirated during any of the (maximum of) nine trials with any of the three consistencies, he or she was considered an aspirating patient in this study. All patients were categorised either as aspirating or not.

### Statistical analysis

Logistic regression was used to predict the outcome (dichotomous variable: aspiration present/absent). Model performance was evaluated by constructing receiver operating characteristics (ROC) curves and computing areas under the curve (AUC). To objectively evaluate whether individual questionnaire items contributed to prediction, a penalised version of logistic regression was used on all items. To that end, a variant of the LASSO regression was applied, namely the elastic net. LASSO allows to simultaneously perform model selection and estimation, whereby variables not contributing to prediction are removed from the model. A penalty parameter determines how many variables are retained, which was chosen automatically by a cross-validation procedure. To evaluate internal consistency, we again applied cross-validation procedure. To this end, the data set was subdivided into 55 pieces (corresponding to a leave-two-out) and in turn each piece was left out of the data set one-by-one. The models described above (logistic regression, LASSO) were fitted on the remaining data and the models were applied to the left-out piece. This procedure ensures that data for which outcomes are predicted have not been used in the model fitting. After completing the 55 rounds of model fitting, each individual has been predicted once and the resulting predictions can be used to generate ROC curves and the corresponding AUC. R version 3.4.0 R version 3.5.0 and packages *AUC* and *glmnet* were used for all analyses [29]. All statistical testing and interpretations were performed by an experienced statistician (S.B.).

## Results

### Patient characteristics

One-hundred eleven patients were included from June 2014 till November 2015 (Table 1). All patients agreed to participate in the current study. No patients were excluded due to cognitive impairments. Sixty-seven subjects (60%) were male with a median age of 65 years (IQR 58–71) compared to 44 (40%) female subjects with a median age of 67 years (IQR 52–74). Medical diagnoses included head and neck cancer (36%) and neurological disorders (37%) such as stroke, Parkinson’s disease, multiple sclerosis and myotonic dystrophy. The remaining patients (27%) had diagnoses like general weakness due to other diseases, cricopharyngeal muscle hypertrophy or epiglottitis. The median FOIS score for the total group was 6 (IQR 4–7), so most patients had an oral intake with some restrictions.

**Table 1:** Subject characteristics

Number of subjects [*n* (%)]	Total group	111
	Male (%)	67 (60)
	Female (%)	44 (40)
Age in years [Med (IQR)]	Total group	66 (56–72)
	Male	65 (58–71)
	Female	67 (52–74)
Medical diagnosis [*n* (%)]	Head and neck cancer	40 (36)
	Neurological disorder	41 (37)
	Other	30 (27)
FOIS^1^ [Med (IQR)]		6 (4–7)

^1^Range 1–7: ‘Nothing by mouth’ to ‘Total oral diet with no restrictions’

### Descriptive statistics

Table 2 displays the descriptive statistics for the SWAL-QOL, DHI, VAS and EAT-10. FEES showed no aspiration in any of the nine trials using three different viscosities (thin, thick, solid consistency) in 90 (81%) patients. A group of 21 (19%) patients aspirated on at least one swallow trial.

**Table 2:** Descriptive analysis of patient self-evaluation questionnaires (*n* = 111): SWAL-QOL, Dysphagia Handicap Index (DHI), Visual Analogue Scales (VAS), and EAT-10

Questionnaire1	(Sub)scale	Range scale	Median (IQR)
SWAL-QOL	Burden	0–100	38 (13–75)
	Eating duration	0–100	38 (0–75)
	Eating desire	0–100	67 (33–92)
	Food selection	0–100	63 (25–75)
	Communication	0–100	75 (38–88)
	Fear	0–100	75 (56–94
	Mental health	0–100	55 (35–85)
	Social functioning	0–100	55 (30–85)
	Fatigue	0–100	58 (33–75)
	Sleep	0–100	75 (38–88)
	Symptom score	0–100	61 (46–71)
DHI	Physical	0–36	16 (10–22)
	Functional	0–36	20 (10–28)
	Emotional	0–28	10 (4–18)
	Total score	0–100	48 (28–64)
	Severity question	1–7	5 (4–6)
VAS	Severity (FHS)	0–100	51 (30–80)
	Impairment (HR-QoL)	0–100	55 (30–85)
EAT-10	Total score	0–40	15 (8–23)

^1^Higher scores indicate higher degree of disability

### Prediction modelling

To build prediction models, first two sets of variables (A and B) were specified a priori (Table 3). In a second, exploratory step, automatic variable selection was used to choose the prediction model.

**Table 3:** Overview of included variables and results per prediction model

	Model 1						Model 2			Model 3 (LASSO)					
	Set A (Fig. 1a) AUC 0.862			Set B (Fig. 1b) AUC 0.852			Set A (Fig. 1c) AUC 0.874			Set A (Fig. 1d) AUC 0.922			Set B (Fig. 1e) AUC 0.915		
	OR	95% CI	*P* value	OR	95% CI	*P* value	OR	95% CI	*P* value	OR	95% CI	*P* value	OR	95% CI	*P* value
(Intercept)	NA	NA	0.40	NA	NA	0.07	NA	NA	0.52	NA	NA	1.00	NA	NA	1.00
SWAL-QOL burden	0.99	0.96–1.02	0.39				0.99	0.96–1.01	0.31	1.00	0.97–1.03	0.96			
SWAL-QOL Food selection	1.02	0.99–1.05	0.14	1.02	1–1.04	0.06	1.03	1.00–1.05	0.08	1.03	1.00–1.07	0.05	1.04	1.00–1.07	0.03
SWAL-QOL Eating duration	1.01	0.98–1.03	0.67				1.00	0.97–1.03	0.97	0.98	0.94–1.01	0.21			
SWAL-QOL Eating desire	1.00	0.98–1.02	0.96				1.00	0.98–1.02	0.98	1.01	0.98–1.04	0.57			
SWAL-QOL Symptom	0.99	0.94–1.03	0.57				0.82	0.66–1.01	0.06	0.81	0.61–1.07	0.14			
SWAL-QOL Symptom squared							1.00	1.00–1.00	0.06	1.00	1.00–1.00	0.10	1.00	1.00–1.00	0.86
DHI subscale physical	0.95	0.86–1.05	0.33	0.96	0.87–1.05	0.34	0.95	0.86–1.05	0.31				0.90	0.80–1.02	0.09
DHI subscale functional	1.04	0.94–1.16	0.41	1.05	0.96–1.15	0.29	1.04	0.94–1.16	0.44				1.11	0.99–1.24	0.08
DHI subscale emotional	1.03	0.91–1.15	0.67	1.07	0.97–1.18	0.17	1.01	0.89–1.13	0.92				1.02	0.91–1.14	0.72
DHI item 1p^1^ score 2										1.96	0.28–13.98	0.50	2.79	0.41–19.11	0.30
DHI item 1p^1^ score 4										34.90	1.91–638.76	0.02	46.57	2.87–756.22	0.01
DHI severity question^2^ score 2										0.00	0.00-INF	1.00	0.00	0.00-INF	1.00
DHI severity question^2^ score 3										0.00	0.00-INF	1.00	0.00	0.00-INF	1.00
DHI severity question^2^ score 4										0.00	0.00-INF	1.00	0.00	0.00-INF	1.00
DHI severity question^2^ score 5										0.00	0.00-INF	1.00	0.00	0.00-INF	1.00
DHI severity question^2^ score 6										0.00	0.00-INF	1.00	0.00	0.00-INF	1.00
DHI severity question^2^ score 7										0.00	0.00-INF	1.00	0.00	0.00-INF	1.00
VAS severity	0.99	0.96–1.03	0.76				0.99	0.95–1.03	0.73	0.96	0.91–1.02	0.21			
VAS impairment	1.01	0.98–1.05	0.45				1.02	0.98–1.06	0.44	1.04	0.99–1.10	0.14			
EAT10 item 9^3^ score 1	33.87	3.70–310.01	0.00	30.25	3.78–241.77	0.00	51.78	4.74–565.29	0.00	100.41	3.05–3300.58	0.01	93.39	2.88–3026.65	0.01
EAT10 item 9^3^ score 2	15.82	1.62–154.72	0.02	16.05	1.86–138.56	0.01	28.15	2.29–346.29	0.01	74.17	1.67–3289.54	0.03	90.10	2.08–3899.97	0.02
EAT10 item 9^3^ score3	47.62	4.42–513.04	0.00	51.11	5.42–481.70	0.00	80.10	5.83–1100.82	0.00	36.75	0.98–1380.57	0.05	68.33	1.78–2621.67	0.02
EAT10 item 9^3^ score 4	26.80	2.41–298.47	0.01	33.04	3.39–321.76	0.00	35.89	2.75–469.06	0.01	6.67	0.16–280.41	0.32	6.48	0.21–200.31	0.29
FOIS	0.72	0.49–1.07	0.11	0.70	0.48–1.02	0.06	0.69	0.45–1.06	0.09	0.67	0.43–1.03	0.07	0.81	0.52–1.26	0.35

^1^DHI item 1p: cough when I drink (score 1–3: never, sometimes, always)

^2^DHI: Patient self-rated severity of dysphagia (score: 0–7; normal to severe problem)

^3^EAT-10 item 9: I cough when I drink (score: 0–4; no problems—severe problems)

**Fig. 1:**
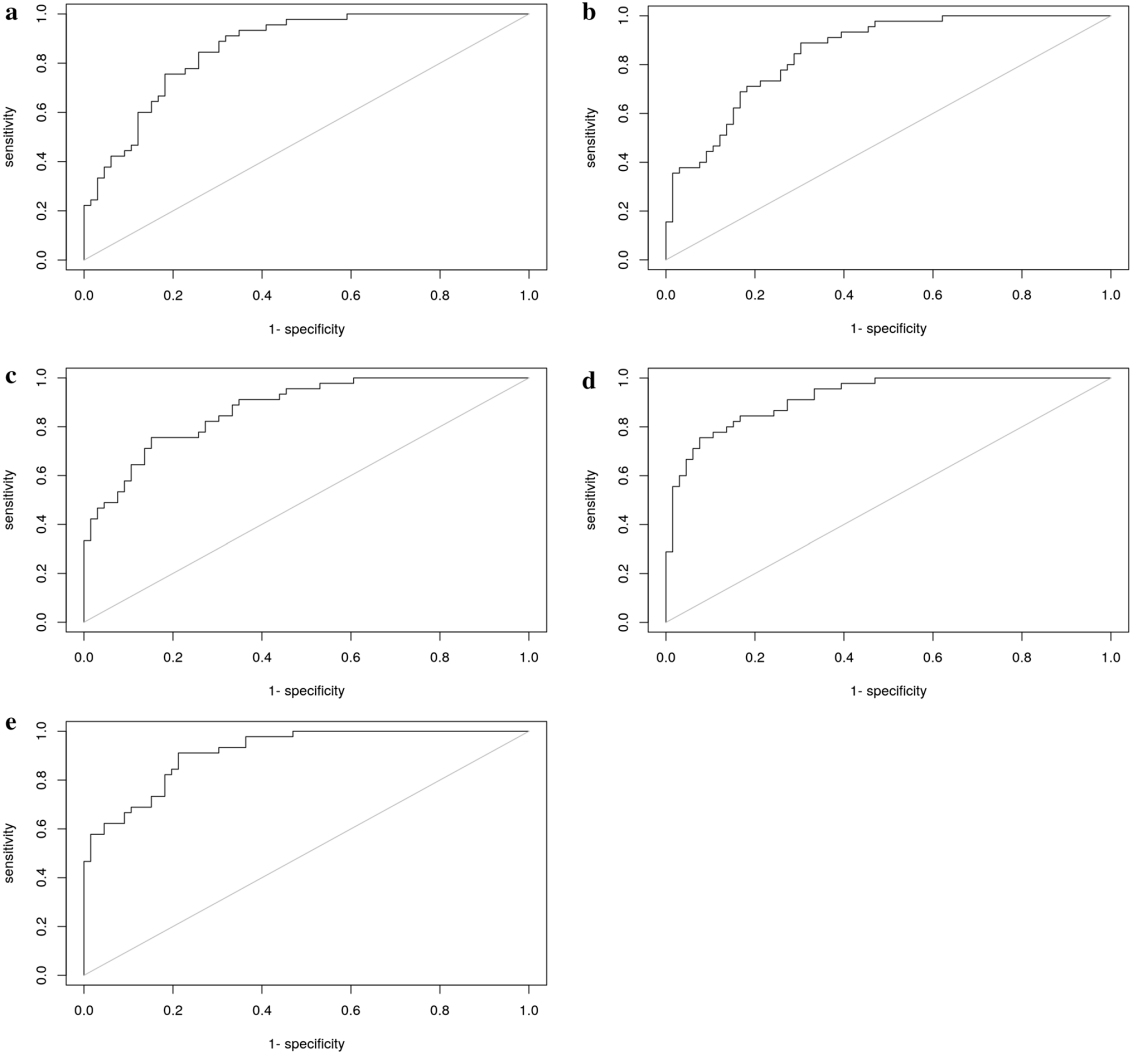
**a** ROC Model 1 Variable set (A) included the following variables: SWAL-QOL subscales Burden, Food selection, Eat duration, Eat desire and Symptom scale, DHI subscales (Functional, Physical and Emotional), VAS scales (Severity and Impairment), EAT-10 item 9 (‘I cough when I eat’) and FOIS. AUC 0.862. **b** ROC Model 1 Variable set (B). Reduced model to SWAL-QOL Food Selection, DHI subscales (Functional, Physical and Emotional), EAT-10 item 9 (‘I cough when I eat’) and FOIS. AUC 0.852. **c** ROC Model 2 Variable set (A), included the following variables: SWAL-QOL subscales Burden, Food selection, Eat duration, Eat desire, Symptom scale and Symptom scale squared, DHI subscales (Functional, Physical and Emotional), VAS scales (Severity and Impairment), EAT-10 item 9 (‘I cough when I eat’) and FOIS. AUC 0.874. **d** ROC Model 3 Variable set (A) included the following variables: SWAL-QOL subscales Burden, Food selection, Eat duration, Eat desire, Symptom scale and Symptom scale squared, DHI item 1p (‘I cough when I drink’), DHI Severity Question, VAS scales (Severity and Impairment), EAT-10 item 9 (‘I cough when I eat’) and FOIS. AUC 0.922. **e** ROC Model 3 Variable set (B) included the following variables: SWAL-QOL subscales Food selection and the Symptom scale squared, DHI subscales (Functional, Physical and Emotional), DHI item 1p (‘I cough when I drink’), DHI Severity question, EAT-10 item 9 (‘I cough when I eat’) and FOIS. AUC 0.915

#### Pre-specified variables

The first set of variables (A) in the logistic regression model was selected on the basis of the literature (including systematic and psychometric reviews on FHS and HR-QoL measures in OD) [7, 20, 30] and clinical experience. Six subscales of the SWAL-QOL (HR-QoL) were considered of lesser importance due to low correlations with oropharyngeal dysphagia and, therefore, excluded: Communication, Fear, Mental health, Social functioning, Fatigue and Sleep [31]. The remaining subscales, namely Burden, Food selection, Eating duration and Eating desire, and the Symptom scale of the SWAL-QOL were included, as were the DHI total subscale scores (Functional, Physical and Emotional subscales) and both VAS scales on swallowing (Severity and Impairment) [23]. As coughing is considered a clinically relevant symptom of aspiration, item 9 of the EAT-10 (I cough when I eat) was also listed [32]. Lastly, the FOIS was added to include information about oral intake. This prediction model yielded an AUC of 0.862. The cross-validated AUC was 0.736.

The second set (B) included fewer variables: the subscale food selection of the SWAL-QOL, the DHI subscales (Functional, Physical and Emotional), item 9 of the EAT-10 and the FOIS. Both VAS scales and the remaining SWAL-QOL subscales were excluded. This reduced model obtained an AUC of 0.852. The cross-validated AUC was 0.775.

The first analysis using set A identified a non-linear association between the gold standard and the Symptom scale of the SWAL-QOL, which led to the inclusion of a quadratic term for the Symptom scale score. Based on this second model, an AUC of 0.874 was found. Because the Symptom scale score was not included in set B, this prediction model remained unchanged.

#### Automatic variable selection

The first penalised logistic regression included all available variables. Based on this regression, certain variables were added to sets A (model 2) and B (model 1). For the first set of variables (A), the DHI additional question on severity (Severity question) and the DHI item 1p (‘I cough when I drink’) were added. For the second set (B), the squared Symptom scale score was added in addition to the two DHI items (Severity question and item 1p). This yielded AUCs of 0.922 for set A and 0.915 for set B. The cross-validated AUC for set A was 0.770.

Table 3 provides an overview of the included variables and the results per prediction model by showing per item the odds ratio, 95% confidence interval and *p* value. Figure 1a-e presents ROC figures and AUC outcomes per model. The formula, as shown below, predicts the presence of aspiration in an individual subject based on the penalised logistic regression model (model 3) using the first set of variables A with an AUC of 0.922. The sensitivity and specificity were, respectively, 90% and 72.7%; the PPV of 68.7% and NPV of 90.6%. The inverse logistic function of the final score *X* indicates the chance of aspiration: *P*_aspiration_*f*(*x*) = 1/(1 + exp(−*x*)).
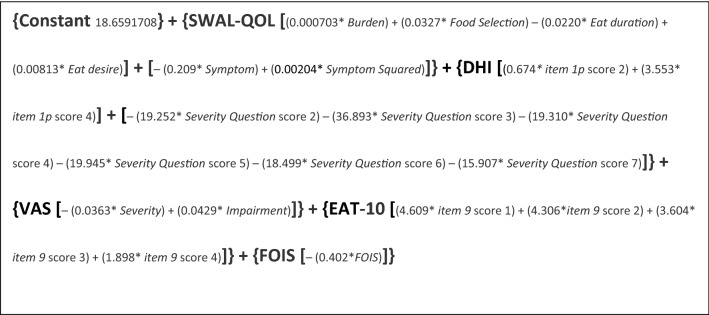


Thus, to determine the chance of aspiration in an individual with OD, the following scores need to be entered in the formula:

- SWAL-QOL: subscales Burden, Food Selection, Eat Duration, Eat Desire and Symptom score (ranging from 0 to 100);
- DHI: Item 1p and Severity question. In the formula variables are included per score for item 1p (score 0, 2 or 4) and for Severity question (score 2 to 7; not 1). These variables include binary numbers (present = 1; absent = 0); For example, if item 1p is scored 2, the section of the formula relating to item 1p is completed as follows: (0.6740265*1) + (3.5526187* 0);
- VAS: Severity and Impairment (score 0–100);
- EAT10: item 9. In the formula, variables are included per score for item 9 (score 1, 2, 3 or 4). These variables are expressed as binary numbers (present = 1; absent = 0);
- FOIS (score 0–7).

Next, to determine the chance of aspiration, the inverse logistic function of the final score *X* needs to be calculated.

## Discussion

The purpose of this study was to build a prediction model for aspiration in patients with OD using common self-evaluation questionnaires and patients' oral intake status. Logistic regression modelling is the preferred method for this [33]. Herein, the number of parameters tested determines the size of the study population needed [34]. Both clinical experience and prior knowledge from the literature may be used to limit the number of predictors in such models. The variable selection was based on these assumptions. Good accuracy was found [35], i.e., AUC of 0.86 and 0.85 for variable sets A and B, respectively. Subsequently, LASSO regression showed excellent accuracy, i.e., AUC of 0.92 for selection of variables from both sets A and B. The resulting formula may be used in the future as a guide to predict aspiration in patients with OD. Our evaluation of internal consistency using cross-validation show that AUCs to be expected in repeat studies are somewhat lower and range between 0.74 and 0.77. The fact is that the smallest, predefined set of variables (B) resulted in the highest cross-validated AUC. This indicates that the relatively small sample size limits the number of predictors that can be used. The cross-validated accuracy for the set B and the LASSO model was almost identical (0.77), indicating that the LASSO is a viable alternative to logistic regression.

This study has some limitations. First, the subjects had high FOIS scores, i.e., no functional impairments in oral intake or only mild impairments. This may have influenced our findings and the generalizability of the results. In view of that possibility, a consecutive series of patients was included to avoid selection bias. As such, our population forms a representative sample of persons visiting an outpatient clinic for dysphagia in an academic hospital setting. Second, only details on the LASSO regression model using the set of variables A are presented here. However, as the AUCs were almost equal for both sets (A and B) when using LASSO regression modelling, the option of using set B in daily clinics might be considered as well. Clinicians may prefer to use DHI subscales rather than adding the SWAL-QoL subscales and VAS scales on Severity and Impairment. Future studies may consider such clinical preferences when building regression models. Third, as self-report measures target patient populations with adequate comprehensive reading skills, self-report measures may not be appropriate for patient populations with severe cognitive deficits such as persons with dementia or acquired brain injury. Still, self-report is considered an important part of the multidimensional assessment of dysphagia [3, 36] and, therefore, should be included in the assessment of OD when possible. Fourth, to reduce patient-experienced burden of assessment and facilitate data collection, patients in the waiting room may be asked to complete all self-report items directly into a computer or digitized system (e.g., mobile phone app or web application) while waiting for their clinician’s appointment, after which the chance of aspiration based on the final predictive model will be determined automatically. Further, the use of computerized data collection may support implementation of the predictive model in daily clinical practice; when implementing the model, only selected items need to be included to save time and reduce patient burden: five (out of eleven) SWAL-QoL subscales plus six other items (from different questionnaires). Fifth, external validation of the findings in another group of patients with OD was not performed. Nonetheless, all predictive models showed good to excellent accuracy with all AUC ≥ 0.85 and AUC exceeding 0.92 when using LASSO regression. Even though no great differences are expected when other groups of patients with OD are included, it may be interesting to compare the current results based on a patient population showing diversity in underlying medical conditions (e.g., head and neck cancer, neurological disorders such as stoke and Parkinson’s disease), with those from future studies in more homogeneous populations with OD. Casting the net wider might reveal similarities or discrepancies in study outcomes; to what degree our predictive model can be generalised to other patient populations remains to be evaluated in follow-up research. Our model can be considered a first step towards the assessment of aspiration risk in patients with OD using oral intake and self-report questionnaires only. The high accuracy of the final prediction model seems to make this a very promising avenue.

These findings are relevant for clinical practice and underscore the importance of self-reported evaluations in the clinical assessment of patients with OD. Until now, these questionnaires were used to measure concepts such as FHS and HR-QoL. They were not used for decision-making; specifically, they were not used to determine whether a patient with dysphagia was at risk for aspiration. The current study suggests that in the absence of gold standard measures, an accurate risk assessment can be performed on the grounds of combined oral intake and self-reported FHS and HR-QoL. This predictive model determines the chance of aspiration, not just a ‘pass or fail’ outcome, with excellent performance (AUC 0.92). This shows the additional value compared to a screening for dysphagia such as water swallow test with a nominal outcome (pass/fail). Possibly, future studies may address the usefulness of the current assessment protocol in clinical settings such as nursing homes or general practices where access to VFSS or FEES may still be limited, in contrast to the widespread availability in tertiary centres nowadays. Automated calculation using a web-based application or mobile app will improve feasibility of the model. In addition, to increase the robustness of the model, reducing the number of variables should be studied, however, at the cost of predictive performance. Furthermore, the use of a non-instrumental assessment protocol to identify aspiration in patients with OD may reduce costs in healthcare.

## Conclusions

This study shows that aspiration in patients with OD may be predicted by a cost-effective, simple and non-invasive assessment protocol including oral intake status and patient self-evaluation questionnaires on FHS and HR-QoL. A predictive model was built using data from a consecutive series of patients at an outpatient clinic of a tertiary care centre. This model may be used to predict aspiration in patients with OD.
